# Supplementary material for: Coordination of alternative splicing and alternative polyadenylation revealed by targeted long read sequencing
Source: Nat Commun. 2023 Sep 7;14:5506. doi: 10.1038/s41467-023-41207-8 (PMC10484994; doi:10.1038/s41467-023-41207-8)
Supplement: Supplementary file 1 — Supplementary Information [file 41467_2023_41207_MOESM1_ESM.pdf]

Supplementary Figures

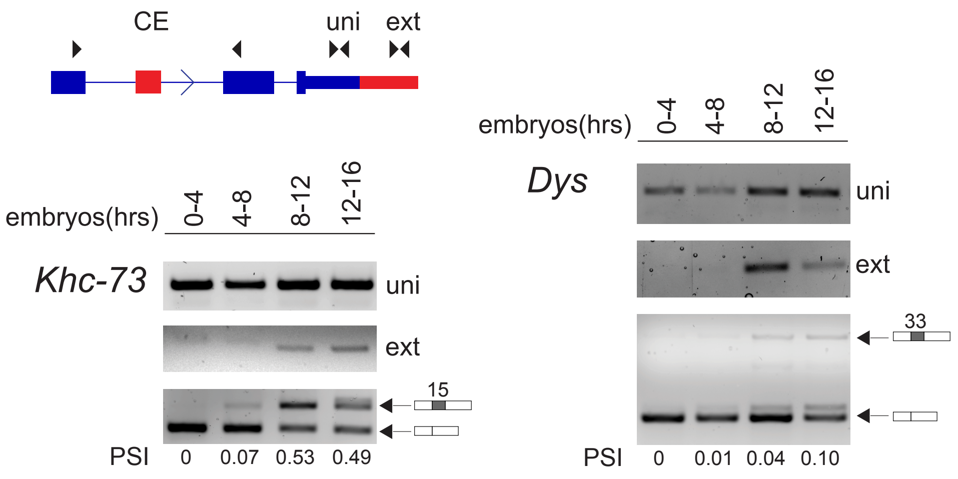

Supplementary Figure 1. RT-PCR confirms trend of coordinated CE splicing and 3'UTR lengthening during embryonic development. RT-PCR data for *Khc-73* and *Dys* shown. Source data are provided as a source data file.

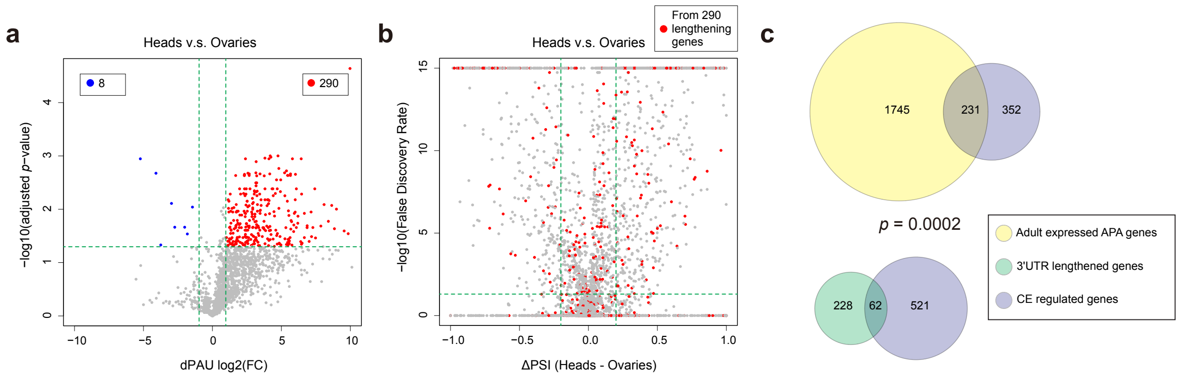

Supplementary Figure 2. CE AS is associated with 3'UTR lengthening in adult heads. (**a**) Short read RNA-Seq analysis of adult head versus ovary shows that 290 genes exhibit significant 3'UTR lengthening. n=3 biologically independent samples. *p* values are calculated from two-tailed t-test, and then adjusted using FDR. (**b**) Distribution of PSI change (PSI (heads) – PSI (ovaries)) of adult head vs ovary samples. From the 290 3'UTR lengthening genes (red dots), 63 significant CE skipping and 127 inclusion events are revealed. (**c**) In adult head vs ovary samples, 3'UTR lengthening genes are significantly associated with regulated CE events (two-sided Fisher's exact test, *p*=0.002). Horizontal dashed lines in (a) indicate adjusted *p*=0.05, and in (b) indicate FDR=0.05. Vertical dashed lines in (a) indicate FC=0.5 (left) and FC=2 (right), while in (b) indicate ΔPSI=-0.2 (left) and ΔPSI=0.2 (right).

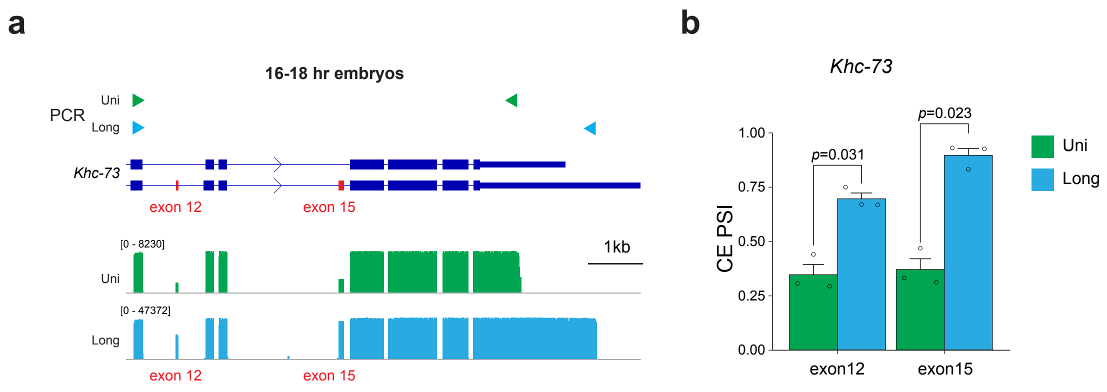

Supplementary Figure 3. Resolving CE splicing patterns between 3'UTR isoforms using targeted PCR based Nanopore sequencing. (**a**) RT-PCR amplification strategy for performing Nanopore sequencing of targeted region of *Khc-73* (top). Coverage plot of long-reads are shown corresponding to the amplicons that cover all isoforms (Uni) or long 3'UTR isoform specifically (Long). (**b**) Bar plot representing PSI calculated from Nanopore long-read sequencing of long and uni samples. Mean+SEM is shown, n=3 biologically independent samples. Two-tailed paired t-test. Source data are provided as a source data file.

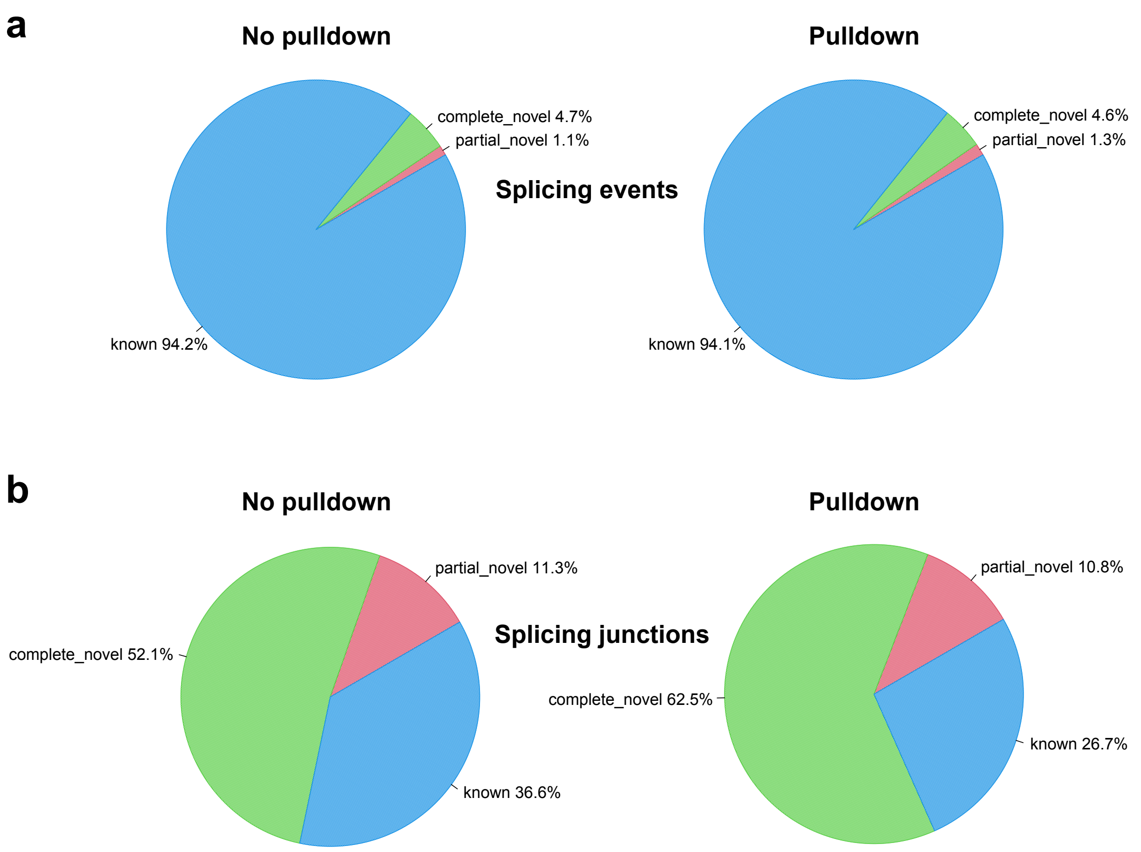

Supplementary Figure 4. Distribution of splicing events detected from PL-Seq. Pie charts displaying read distribution for (**a**) Splicing events and (**b**) Splicing junctions for “No pulldown” and “Pulldown” libraries. Reads are characterized as complete novel, partial novel or known according to the fly Ensembl dm6 annotation.

Gene	CE	16-18 hr embryos: PSI (Long - Short)	16-18 hr embryos: *p* Value (Long v.s. Short)	16-18 hr embryos: Coordinated AS-APA (Short-read RNA-Seq)	Adult heads: PSI (Long - Short)	Adult heads: *p* Value (Long v.s. Short)	Adult heads: Coordinated AS-APA (Short-read RNA-Seq)
Dys	33	0.969 - 0.017	3.736E-07	Yes	0.987 - 0.031	1.061E-04	No
Khc−73	15	0.968 - 0.023	1.002E-06	Yes	0.888 - 0.056	6.831E-04	Yes
pod1	9	0.703 - 0.105	4.259E-04	No	0.515 - 0.529	8.863E-01	Yes
X11L	8	0.629 - 0.095	8.225E-04	Yes	0.467 - 0.065	9.014E-04	Yes
spen	18	0.781 - 0.257	4.735E-04	No	0.941 - 0.425	7.990E-04	Yes
CASK	17	0.970 - 0.515	5.049E-02	Yes	0.867 - 0.441	1.337E-01	Yes
Mbs	11	0.690 - 0.368	1.487E-02	Yes	0.665 - 0.622	2.380E-01	Yes
mtd	12	0.325 - 0.024	1.287E-01	No	0.897 - 0.389	1.035E-03	Yes
nuf	9	0.347 - 0.061	3.077E-05	Yes	0.729 - 0.668	6.813E-01	Yes
CG8671	10	0.292 - 0.023	3.432E-03	No	0.460 - 0.130	1.258E-02	Yes
tutl	15	0.870 - 0.644	1.819E-02	Yes	1.000 - 0.958	1.150E-01	No
Camta	9	0.191 - 0.000	3.583E-02	No	N/A	N/A	Yes
par−1	13	0.256 - 0.074	5.857E-03	Yes	0.227 - 0.047	1.749E-03	Yes
Evi5	2	0.265 - 0.117	2.061E-01	Yes	N/A	N/A	Yes
shi	10	0.162 - 0.033	1.919E-04	No	0.635 - 0.440	8.503E-03	Yes
CdGAPr	4	0.248 - 0.124	5.387E-01	No	0.315 - 0.301	8.133E-01	Yes
Ptp10D	12	0.112 - 0.003	1.113E-02	Yes	0.113 - 0.000	4.200E-02	Yes
Nc73EF	15	0.066 - 0.031	6.105E-02	Yes	0.051 - 0.152	1.246E-01	Yes
gish	14	0.680 - 0.654	4.117E-01	Yes	0.828 - 0.601	1.468E-02	Yes
Vav	12	0.971 - 0.957	8.069E-01	No	0.936 - 1.000	9.377E-02	Yes
stai	6	0.859 - 0.887	1.615E-02	Yes	0.115 - 0.298	1.425E-02	Yes
CG15312	6	0.007 - 0.064	8.190E-02	No	0.657 - 0.747	1.610E-02	Yes
Crag	10	0.654 - 0.868	4.866E-02	No	0.382 - 0.444	2.218E-01	Yes
lap	15	0.285 - 0.728	8.988E-05	Yes	0.272 - 0.546	1.253E-02	Yes
Eip63E	4	0.355 - 0.897	3.306E-05	No	0.306 - 0.912	2.294E-04	Yes
Calx	4	0.033 - 0.591	3.714E-05	No	0.250 - 0.206	6.125E-01	Yes
Lar	16	0.100 - 0.774	1.395E-02	Yes	N/A	N/A	Yes
Dscam1	23	0.025 - 0.811	1.543E-04	Yes	0.726 - 0.847	2.874E-02	No

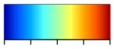

PSI (Long - Short)

PL-Seq Verified Linked AS and APA

Supplementary Figure 5. Summary of PSI differences between long 3'UTR and short 3'UTR isoforms. CE splicing difference derived from PL-Seq analysis is shown as PSI (long – short) by heatmap. Individual values of PSI from Long 3'UTR and Short 3'UTR isoforms are listed in the colored cells. *p* values from two-tailed paired t-test using PL-Seq data are shown. Purple coloring indicates a significant difference in the t-test. "Coordinated AS-APA" (“Yes” or “No”) refers to whether the gene was determined to exhibit regulated AS and APA from short read RNA-Seq analysis (Fig. 1d for Late vs Early stage embryos, and Supplementary Fig. 2b for Heads vs Ovaries).

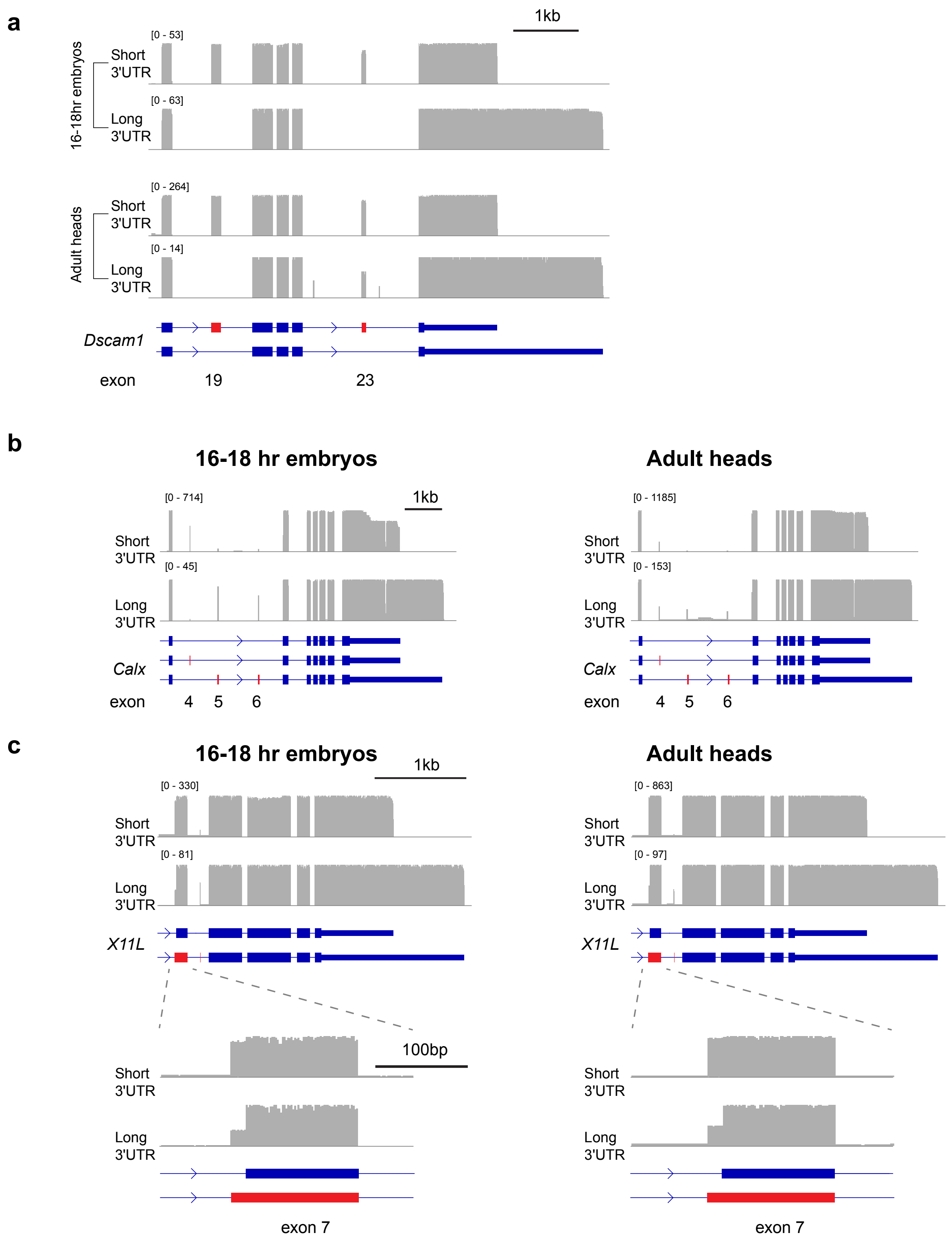

a
1kb
16-18hr embryos
Short 3'UTR
[0 - 53]
Long 3'UTR
[0 - 63]
Adult heads
Short 3'UTR
[0 - 264]
Long 3'UTR
[0 - 14]
Dscam1
exon
19
23
b
16-18 hr embryos
Adult heads
[0 - 714]
1kb
Short 3'UTR
[0 - 45]
Long 3'UTR
Calx
exon
4
5
6
[0 - 1185]
[0 - 153]
c
16-18 hr embryos
Adult heads
1kb
[0 - 330]
[0 - 81]
X11L
[0 - 863]
[0 - 97]
100bp
exon 7

Supplementary Figure 6. Additional PL-Seq data tracks of genes exhibiting 3'UTR connected AS. (**a**) Filtering of reads to detect *Dscam1* exon 19 AS in 16-18 hr embryos and adult heads. (**b**) Coverage tracks of PL-Seq data showing splicing pattern in short and long 3'UTR reads. For *Calx*, exon 4 acts as both a CE and mutually exclusive to exons 5 and 6. (**c**) For *X11L*, exon 8 AS and the usage of an alternative upstream 3' splice site of exon 7 is different between short vs long 3'UTR isoforms.

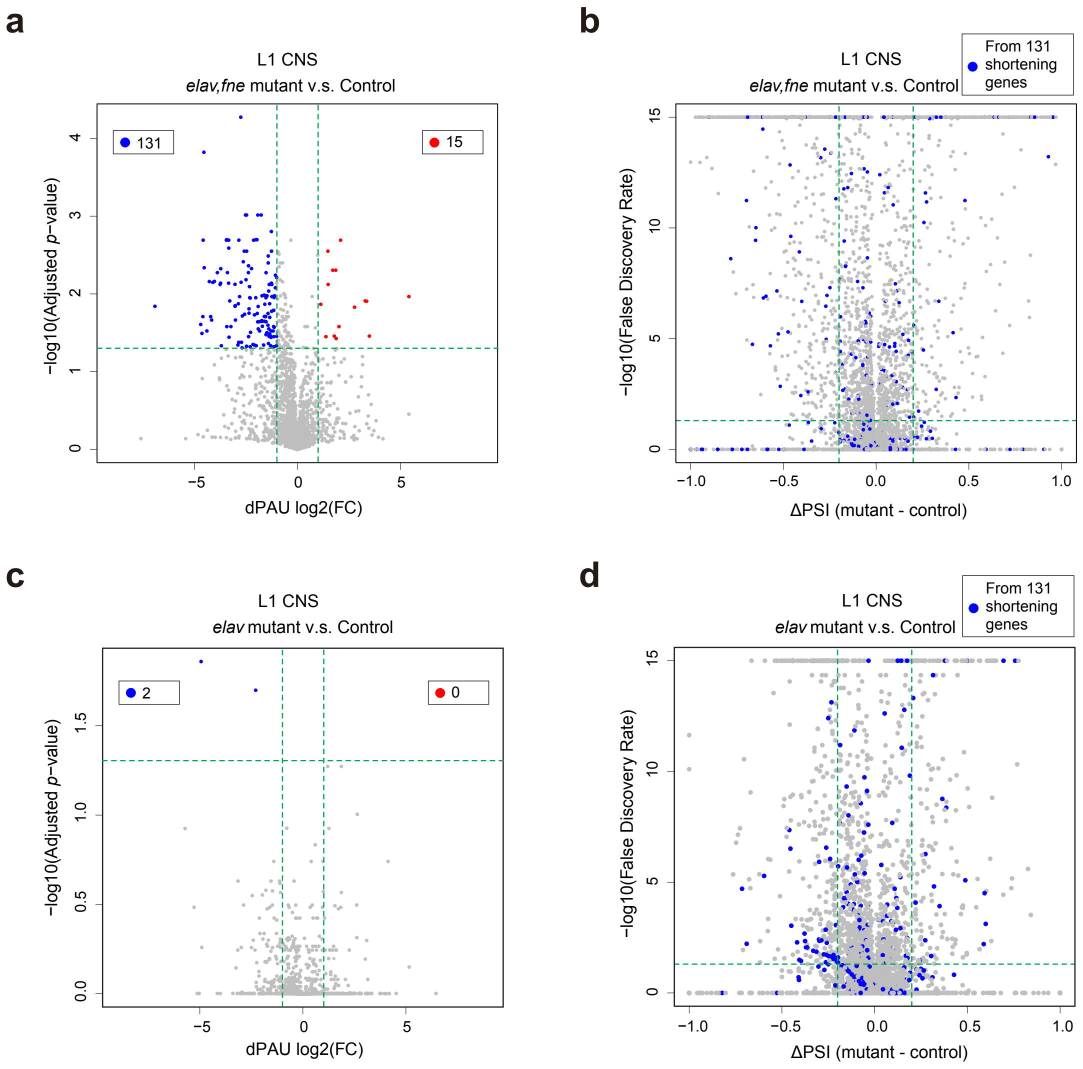

Supplementary Figure 7. Short read RNA-Seq analysis of APA and CE AS in L1 CNS. (**a**) Short read RNA-Seq analysis of dPAU in L1 CNS samples from *elav,fne* mutant vs control using QAPA. 131 genes show significantly shortened 3'UTR in *elav,fne* mutant. n=3 biologically independent samples. *p* values are calculated from two-tailed t-test, and then adjusted using FDR. (**b**) Change of CE events in *elav,fne* mutants compared to control. The CE events from 131 3'UTR shortened genes from panel (**a**) are shown in blue. (**c**) Short read RNA-Seq analysis of dPAU in L1 CNS samples from *elav* mutant vs control using QAPA. 2 genes show significantly shortened 3'UTR in *elav* mutant. n=3 (CS control) and 2 (*elav* mutant) biologically independent samples. *p* values are calculated from two-tailed t-test, and then adjusted using FDR. (**d**) Change of CE

events in *elav* mutants compared to control. The CE events from 131 3'UTR shortened genes from panel (**a**) are shown in blue. Horizontal dashed lines in (a) and (c) indicate adjusted p=0.05, while in (b) and (d) indicate FDR=0.05. Vertical dashed lines in (a) and (c) indicate FC=0.5 (left) and FC=2 (right), while in (b) and (d) indicate ΔPSI=-0.2 (left) and ΔPSI=0.2 (right).

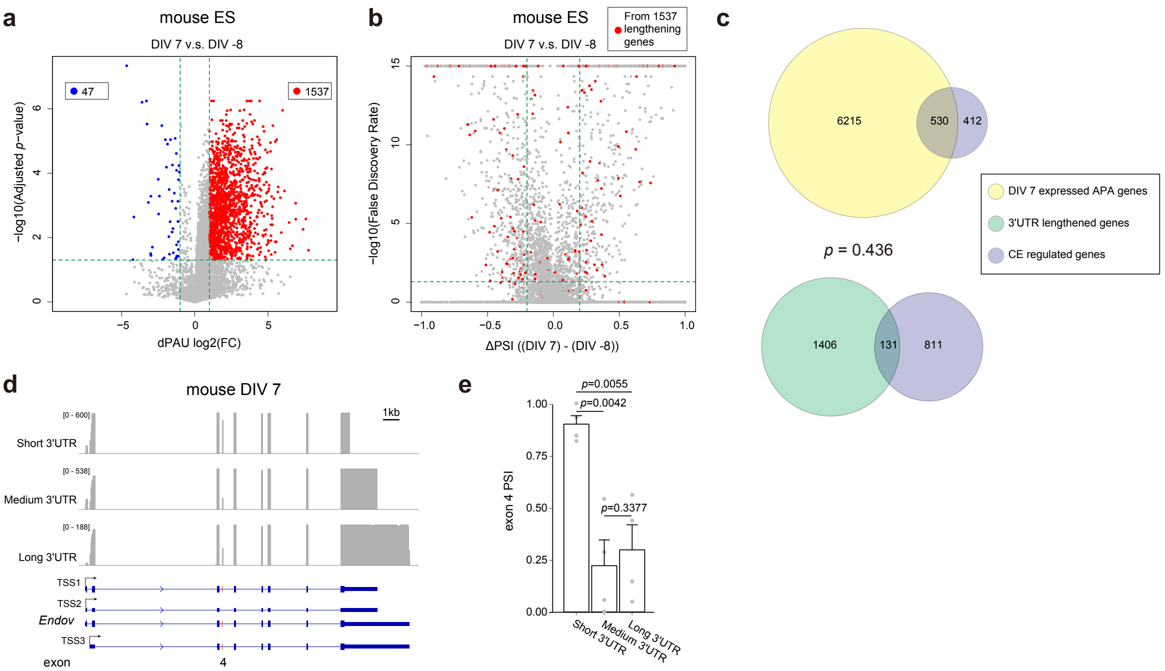

Supplementary Figure 8. PL-Seq data exhibits 3'UTR linked CE splicing in mouse ES cell derived neurons. (**a**) Short read RNA-Seq analysis of mouse ES cell derived neurons (DIV 7) versus undifferentiated ES cells (DIV -8) shows that 1537 genes exhibit significant 3'UTR lengthening. n = 4 (DIV -8) and 5 (DIV 7) biologically independent samples. *p* values are calculated from two-tailed t-test, and then adjusted using FDR. (**b**) Distribution of PSI change (PSI (neurons) – PSI (ES cells)). Horizontal dashed lines in (a) indicate adjusted *p*=0.05, and in (b) indicate adjusted FDR=0.05. Vertical dashed lines in (a) indicate FC=0.5 (left) and FC=2 (right), and in (b) indicate ΔPSI=-0.2 (left) and ΔPSI=0.2 (right). (**c**) Fisher's exact test (two-sided) shows that in mouse neurons vs ES samples, 3'UTR lengthening genes are not significantly associated with regulated CE events (*p*=0.624). (**d**) *Endov* exon 4 splicing pattern of short, medium and long 3'UTR reads in mouse ES cell derived neurons (DIV 7) as shown by PL-Seq coverage tracks. (**e**) PL-Seq quantification of *Endov* exon 4 PSI shows that CE PSI is significantly higher in short 3'UTR isoforms when compared to medium and long 3'UTR isoforms in mouse ES cell derived neurons (DIV 7). Two-tailed paired t-test. Data is shown as Mean+SEM. n=4 biologically independent samples. Source data are provided as a source data file.
