# Supplementary material for: Coordination of alternative splicing and alternative polyadenylation revealed by targeted long read sequencing
Source: Nat Commun. 2023 Sep 7;14:5506. doi: 10.1038/s41467-023-41207-8 (PMC10484994; doi:10.1038/s41467-023-41207-8)
Supplement: Supplementary file 3 — Description of Additional Supplementary Files [file 41467_2023_41207_MOESM3_ESM.pdf]

Description of Additional Supplementary Files

File Name: Supplementary Data 1
Description: Short-read RNA-Seq analysis summary for the 252 3'UTR lengthening genes and all regulated CE events from 58 out of 252 3'UTR lengthening genes in 2-4hr and 16-18hr embryos. Two-tailed t-test followed by Benjamini-Hochberg correction, n=3 biologically independent samples.

File Name: Supplementary Data 2
Description: Short-read RNA-Seq analysis summary for the 290 3’UTR lengthening genes and all regulated CE events from 62 out of 290 3'UTR lengthening genes in adult ovaries and heads. Two-tailed t-test followed by Benjamini-Hochberg correction, n = 3 biologically independent samples.

File Name: Supplementary Data 3
Description: Oligonucleotide sequences. Biotinylated probe sets for PL-Seq. 1 and 2, designed for AS-APA genes, Used in Fig. 2, 3, 4, and supplementary Fig. 3, 4, and 5. Probe set 3 is used in Fig. 5 and 6 and probe set 4 in supplementary Fig. 8.

File Name: Supplementary Data 4
Description: Short-read RNA-Seq analysis summary for the 131 3'UTR lengthening genes and all regulated CE events from 29 out of 131 3'UTR lengthening genes in L1 CNS of elav,fne mutant and control samples. Two-tailed t-test followed by Benjamini-Hochberg correction, n = 3 biologically independent samples.

File Name: Supplementary Data 5
Description: PL-Seq verified AS-APA genes reported to have coordinated AS and APA in published short-read RNA-Seq data analysis of elav,fne mutant embryos1 .

File Name: Supplementary Data 6
Description: Short-read RNA-Seq analysis summary for distal polyA site usage and all regulated CE events from 29 3'UTR lengthening genes in L1 CNS of elav mutant and control samples. The 29 genes are from Supplementary Data 6. Two-tailed t-test followed by Benjamini-Hochberg correction, n = 3 (CS control) and 2 (elav mutant) biologically independent samples.

File Name: Supplementary Data 7
Description: Short-read RNA-Seq analysis summary for mouse ES cell derived neuron (DIV 7) and control (DIV -8) samples. The distal polyA site usage of 1537 3'UTR lengthening genes and all regulated CE events from 131 out of 1537 3'UTR lengthening genes are included. Two-tailed t-test followed by Benjamini-Hochberg correction, n = 4 (DIV -8) and 5 (DIV 7) biologically independent samples.

File Name: Supplementary Data 8
Description: Primer sequences and coordinates (dm6).

File Name: Supplementary Data 9
Description: CE number and coordinates for each AS-APA gene that was analyzed in this work.

File Name: Supplementary Data 10
Description: PL-Seq data analysis results showing read counts of CE splicing per 3'UTR isoform and total reads per gene (both inclusion and exclusion).

Supplementary Data References:

1. Carrasco, J. et al. ELAV and FNE Determine Neuronal Transcript Signatures through Exon-Activated Rescue. Mol. Cell 80, 156-163.e6 (2020).
