# Supplementary material for: Coordination of alternative splicing and alternative polyadenylation revealed by targeted long read sequencing
Source: Nat Commun. 2023 Sep 7;14:5506. doi: 10.1038/s41467-023-41207-8 (PMC10484994; doi:10.1038/s41467-023-41207-8)
Supplement: Supplementary file 14 — Reporting Summary [file 41467_2023_41207_MOESM14_ESM.pdf]

nature portfolio

Corresponding author(s): Pedro Miura

Last updated by author(s): 08/09/2023

Reporting Summary

Nature Portfolio wishes to improve the reproducibility of the work that we publish. This form provides structure for consistency and transparency in reporting. For further information on Nature Portfolio policies, see our Editorial Policies and the Editorial Policy Checklist.

Statistics

For all statistical analyses, confirm that the following items are present in the figure legend, table legend, main text, or Methods section.

n/a	Confirmed	
☐	☒	The exact sample size (*n*) for each experimental group/condition, given as a discrete number and unit of measurement
☐	☒	A statement on whether measurements were taken from distinct samples or whether the same sample was measured repeatedly
☐	☒	The statistical test(s) used AND whether they are one- or two-sided<br>*Only common tests should be described solely by name; describe more complex techniques in the Methods section.*
☐	☒	A description of all covariates tested
☐	☒	A description of any assumptions or corrections, such as tests of normality and adjustment for multiple comparisons
☐	☒	A full description of the statistical parameters including central tendency (e.g. means) or other basic estimates (e.g. regression coefficient) AND variation (e.g. standard deviation) or associated estimates of uncertainty (e.g. confidence intervals)
☐	☒	For null hypothesis testing, the test statistic (e.g. *F*, *t*, *r*) with confidence intervals, effect sizes, degrees of freedom and *P* value noted<br>*Give P values as exact values whenever suitable.*
☒	☐	For Bayesian analysis, information on the choice of priors and Markov chain Monte Carlo settings
☒	☐	For hierarchical and complex designs, identification of the appropriate level for tests and full reporting of outcomes
☒	☐	Estimates of effect sizes (e.g. Cohen's *d*, Pearson's *r*), indicating how they were calculated

Our web collection on statistics for biologists contains articles on many of the points above.

Software and code

Policy information about availability of computer code

Data collection	MinKNOW (20.10.3-21.06.13) was used to collect Nanopore sequencing data.
Data analysis	NanoPack (v1.41.0), RSeQC (v5.0.1), SAMtools (v1.6.0), (minimap2 (v2.17), STAR (2.7), QAPA (1.2.3), rMATS (4.0.2), R(3.6.1 and 4.2.2) were used for analysis of short/long read RNAseq data. Gene Ontology was performed using FlyEnrichR (https://maayanlab.cloud/FlyEnrichr/). Custom scripts used for PL-Seq data filtering are available at: https://github.com/markandtwin/Pull-a-long.

For manuscripts utilizing custom algorithms or software that are central to the research but not yet described in published literature, software must be made available to editors and reviewers. We strongly encourage code deposition in a community repository (e.g. GitHub). See the Nature Portfolio guidelines for submitting code & software for further information.

Data

Policy information about availability of data

All manuscripts must include a data availability statement. This statement should provide the following information, where applicable:

- Accession codes, unique identifiers, or web links for publicly available datasets
- A description of any restrictions on data availability
- For clinical datasets or third party data, please ensure that the statement adheres to our policy

All the sequencing data is deposited as SRA Bioproject PRJNA771049. Previously published data used for re-analysis are accesible as PRJNA75285, GSE155534 and SRP017778.

Human research participants

Policy information about studies involving human research participants and Sex and Gender in Research.

Reporting on sex and gender	N/A
Population characteristics	N/A
Recruitment	N/A
Ethics oversight	N/A

Note that full information on the approval of the study protocol must also be provided in the manuscript.

Field-specific reporting

Please select the one below that is the best fit for your research. If you are not sure, read the appropriate sections before making your selection.

☒ Life sciences ☐ Behavioural & social sciences ☐ Ecological, evolutionary & environmental sciences

For a reference copy of the document with all sections, see nature.com/documents/nr-reporting-summary-flat.pdf

Life sciences study design

All studies must disclose on these points even when the disclosure is negative.

Sample size	3-6 biologically independent samples were used for gene expression analysis except for elav mutant short-read RNAseq (there were only two replicates available in the public dataset), when a minimum of three biological replicates are recommended in the field (Conesa et al, Genome Biology, 2016).
Data exclusions	For PSI calculations, replicates were required to have at least two reads covering the area of interest (from upstream alternative splicing event to 3' end) to be included.
Replication	3-6 biological replicates were tested. For nanopore sequencing, each replicate was sequenced on separate flow cells at different times. For some lowly expressed gene isoforms, some replications didn't pass the data filtering process (minimum two reads) and thus were excluded from downstream analysis.
Randomization	Experimental groups were determined by genotype.
Blinding	Experimenter was not blinded to genotype. Data collected on the MinION was analyzed using scripts applied without bias to sample.

Reporting for specific materials, systems and methods

We require information from authors about some types of materials, experimental systems and methods used in many studies. Here, indicate whether each material, system or method listed is relevant to your study. If you are not sure if a list item applies to your research, read the appropriate section before selecting a response.

Materials & experimental systems

n/a	Involved in the study	
☒	☐	Antibodies
☒	☐	Eukaryotic cell lines
☒	☐	Palaeontology and archaeology
☐	☒	Animals and other organisms
☒	☐	Clinical data
☒	☐	Dual use research of concern

Methods

n/a	Involved in the study	
☒	☐	ChIP-seq
☒	☐	Flow cytometry
☒	☐	MRI-based neuroimaging

Animals and other research organisms

Policy information about studies involving animals; ARRIVE guidelines recommended for reporting animal research, and Sex and Gender in Research

Laboratory animals	Drosophila strains used in this study: wild type control(w1118), Khc-73(DeltaL), Dscam1(DeltaL), Dscam1(DeltaExon19), Khc-73(DeltaExon15), Elav(5). 16-18hr embryos and 1-5 day mixed adult male and females are used in our experiments.
Wild animals	No wild animals are used in this study.
Reporting on sex	Drosophila of mixed sex was used for analysis.
Field-collected samples	No field collected samples were used in the study
Ethics oversight	No ethical approval was required as only Drosophila melanogaster was used in this study.

Note that full information on the approval of the study protocol must also be provided in the manuscript.
